# Direct Injection of Functional Single-Domain Antibodies from *E. coli* into Human Cells

**DOI:** 10.1371/journal.pone.0015227

**Published:** 2010-12-08

**Authors:** Ana Blanco-Toribio, Serge Muyldermans, Gad Frankel, Luis Ángel Fernández

**Affiliations:** 1 Department of Microbial Biotechnology, Centro Nacional de Biotecnología, Consejo Superior de Investigaciones Científicas (CSIC), Campus Cantoblanco Universidad Autónoma de Madrid (UAM), Madrid, Spain; 2 Laboratory of Cellular and Molecular Immunology, Vrije Universiteit Brussel, Brussels, Belgium; 3 Department of Molecular and Cellular Interactions, Vrije Universiteit Brussel, Brussels, Belgium; 4 Centre for Molecular Microbiology and Infection, Division of Cell and Molecular Biology, Imperial College London, London, United Kingdom; National Institute on Aging, United States of America

## Abstract

Intracellular proteins have a great potential as targets for therapeutic antibodies (Abs) but the plasma membrane prevents access to these antigens. Ab fragments and IgGs are selected and engineered in *E. coli* and this microorganism may be also an ideal vector for their intracellular delivery. In this work we demonstrate that single-domain Ab (sdAbs) can be engineered to be injected into human cells by *E. coli* bacteria carrying molecular syringes assembled by a type III protein secretion system (T3SS). The injected sdAbs accumulate in the cytoplasm of HeLa cells at levels ca. 10^5^–10^6^ molecules per cell and their functionality is shown by the isolation of sdAb-antigen complexes. Injection of sdAbs does not require bacterial invasion or the transfer of genetic material. These results are proof-of-principle for the capacity of *E. coli* bacteria to directly deliver intracellular sdAbs (*intrabodies*) into human cells for analytical and therapeutic purposes.

## Introduction

The ability to express antibody (Ab) fragments in *Escherichia coli* has an enormous biotechnological and therapeutic potential [1]. The smallest Ab fragments (∼12–15 kDa) are the so-called single-domain antibodies (sdAbs), which are composed of a single variable (V) immunoglobulin (Ig) domain [2], [3]. The sdAbs are generated by engineering conventional Igs (e.g. human or murine) [4] or obtained from natural heavy-chain-only Igs expressed by certain animals like camelids [5]. The sdAbs from camelid heavy-chain-only Igs are known as V_HH_ domains or Nanobodies. Importantly, the absence of a paired V domain in V_HH_s does not hinder their affinity for their cognate antigens, which is in the same range of conventional Abs with paired V_H_/V_L_ domains (K_D_∼10^−8^–10^−10^ M). Targets for therapeutic Abs are extracellular including cytokines, matrix proteins, and extracellular domains of membrane receptors [6]. Intracellular proteins (e.g. components of cell signaling cascades) are excellent therapeutic targets but plasma membrane prevents the access of Abs to them. Nonetheless, Ab fragments against different antigens have been expressed intracellularly (intrabodies) as inhibitors of proteins involved, for instance, in carcinogenesis and viral replication [7], [8]. Intrabody expression requires transfer of the encoding gene into the cell, either using transfection with naked DNA, liposomes, or infection with recombinant viral vectors, which raises concerns given its possible integration into the host cell genome. Therefore direct transfer of antibody polypeptides into target cells constitute an attractive alternative. Since *E. coli* is employed for selection, engineering and production of IgGs and Ab fragments [9], [10] this microorganism is an excellent candidate for delivery of intrabodies. Preferably, the delivery system should avoid the use of invasive *E. coli* strains that release their cell content after lysis in the phagosome [11]. Interestingly, intestinal pathogenic *E. coli* strains, such as the enteropathogenic *E. coli* (EPEC) O127:H6 [12] and enterohaemorragic *E. coli* (EHEC) O157:H7 [13], remain extracellular while using a type III protein secretion system (T3SS) to inject specific bacterial proteins, referred to as “effectors”, into mammalian cells [14], [15].

EPEC and EHEC adhere to enterocytes in the gastrointestinal tract while inducing characteristic “attachment and effacement” (A/E) lesions [16]. A chromosomal pathogenicity island of 35–40 kb, called the locus of enterocyte effacement (*LEE*)[17], encodes the proteins responsible of A/E lesion formation, including the outer membrane adhesin intimin (*eae*), the T3SS, and six effectors [18]. T3SSs are supramolecular protein assemblies embedded in the bacterial envelope (called the needle complex or injectisome). They are composed of a cytosolic ATPase, inner and outer membrane rings, a periplasmic shaft and an extracellular needle [19], [20]. Protein translocation is dependent on insertion of hydrophilic translocation pore into the plasma membrane of the eukaryotic cell (the translocon) [19]. In EPEC and EHEC the needle complex is extended up to 700 nm by long and flexible EspA filament of polymerized EspA and the translocon is composed of translocated proteins EspB and EspD [21].

Effectors and translocators, the substrates of T3SSs, contain a non-cleavable N-terminal translocation signal usually comprising the first ∼15–30 residues [20], [22]. N-terminal fusions of effectors with viral antigens and certain enzymes have been secreted through T3SSs for the generation of live vaccines [23], [24] or as translocation reporters [25], [26]. The aim of this study was to determine whether non-invasive *E. coli* bacteria carrying a T3SS can be used to translocate Ab fragments into human cells.

## Results

### Secretion of functional sdAbs into *E. coli* culture media

**Figure 1:**
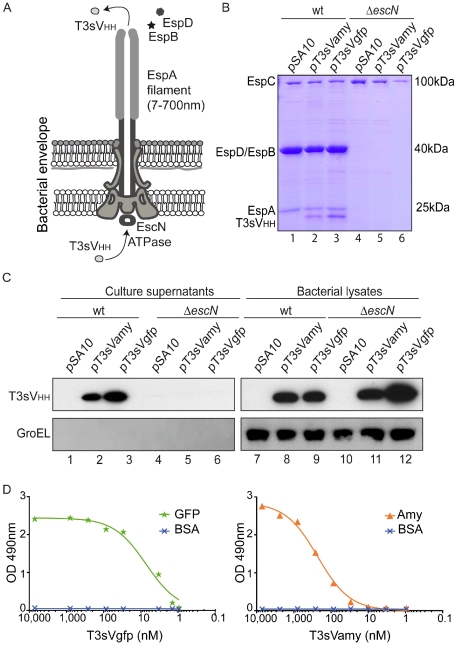
Secretion of sdAbs to the extracellular medium with T3SS of EPEC. (**A**) Schematic representation of the T3SS-complex encoded by EPEC, labeling the essential ATPase EscN, the extracellular EspA filament and the secreted EspB and EspD translocators. The secretion of T3sNbs from the cytoplasm of the bacteria to the extracellular medium is indicated. (**B**) Coomassie staining of TCA-precipitated proteins found in the extracellular media of cultures of wt EPEC or Δ*escN* strains carrying plasmids pSA10, pT3sVamy, or pT3sVgfp, as indicated. Cultures were grown at 37°C in DMEM and induced with 0.1 mM IPTG for 4 h. The protein bands of EspA, EspB, EspD, and that of the Sec-dependent autotransporter EspC, are labeled. Size in kDa of protein standards for SDS-PAGE are shown on the right. (**C**) Western blot analysis of the proteins found in extracellular media (Culture supernantants; lanes 1–6) and cells (bacterial lysates; lanes 7–12) from the same cultures as in (B). WB developed with mAbs anti-E-tag (top panels) or anti-GroEL (bottom panels) to control the absence of cytoplasmic proteins in the extracellular media. (**D**) Binding activity of the secreted sdAbs. ELISA with His-tag purified T3sVgfp (left) and T3sVamy (right), at the indicated concentrations (nM), against their cognate antigens (GFP or Amy) and BSA (negative control). Bound T3sV_HH_s developed with anti-E-tag mAb-POD and their Optical Density (O.D.) determined at 490 nm.

The N-terminal 20 amino acids of the effector EspF, which are fully conserved in EPEC strain E2389/69 and EHEC strain EDL933*stx* (Table 1), were selected to drive the T3 secretion of the sdAb fragments. We chose V_HH_s as sdAb fragments due to their favorable biophysical properties and ability to function as potent enzyme inhibitors [5], [27]. Two characterized V_HH_s, named Vamy and Vgfp, recognizing amylase (Amy) and the green fluorescent protein (GFP) respectively, were employed as models [28], [29]. We used the IPTG-inducible bacterial expresion vector pSA10 (Table 2) to express EspF_20_ T3 signal (T3s) fused to Vamy (T3sVamy) or Vgfp (T3sVgfp) (Fig. S1). The V_HH_s were tagged with His and E-tag epitopes at their C-termini to allow metal-affinity purification and detection with monoclonal antibodies (mAbs). Although T3SS are cell-contact dependent, secretion can be triggered *in vitro* under by growing bacteria under certain growth conditions [20]. In EPEC this is achieved by growth in DMEM 5% CO_2_ at 37°C. Thus, we used this growth conditions to analyze whether T3sV_HH_s are secreted by the T3SS of EPEC (Fig. 1). EPEC wild-type (wt) strain and Δ*escN* strain (Table 1), which lacks the T3SS ATPase EscN [30], transformed with pSA10 (empty vector), pT3sVamy, or pT3sVgfp, were grown in DMEM and induced with IPTG for 3 h. Induction of T3sV_HH_ fusions did not affect the growth of EPEC strains, which reached the same final optical density (OD_600 nm_∼1.2) as cultures with the empty vector. Coomassie staining of proteins secreted from wt EPEC revealed discrete bands corresponding to the T3SS substrates (e.g. EspA, EspB, EspD) and protein bands of ca. 21–23 kDa, corresponding to the expected size of T3sV_HH_s, when bacteria carried pT3sVamy or pT3sVgfp (Fig. 1B; lanes 1–3). No secreted T3sV_HH_s were seen from wt EPEC carrying the empty vector. Neither protein bands corresponding to the T3SS substrates nor the T3sV_HH_s were present in the supernatant of Δ*escN* strain (Fig. 1B; lanes 4–6). A band corresponding to the autotransporter EspC [31], which is secreted by the Sec-pathway, was present in the supernatants of both the wt and Δ*escN* strains.

**Table 1:** *E. coli* strains employed in this study.

*Strain*	*Relevant genotype and features*	*Reference*
*E. coli* JM109	K-12 *λ-, supE44 thi1 mcrA recA1 endA1 hsdR17 gyrA96 relA1 Δlac-proAB, F' (traD36 proAB lacIq lacZ*ΔM15)/cloning strain	[69]
EPEC E2348/69	wild type enteropathogenic EPEC O127:H6	[12]
EPEC Δ*escN*	E2348/69 Δ*escN::Km*/mutant lacking EscN ATPase of the T3SS	[37], [70]
EHEC EDL933*stx*	wild type enterohaemorragic EHEC O157:H7 *stx1- stx2-*	[13], [70]
EHEC Δ*escN*	EDL933*stx* Δ*escN::Km*/mutant lacking EscN ATPase of T3SS	[70]
quad mutant	E2348/69 Δ*eae* Δ*tir* Δ*map* Δ*espF*/attenuated strain	[34]

**Table 2:** Plasmids employed in this study.

*Name*	*Relevant features and application*	*Reference*
pSA10	Ap^r^, pUC-ori, *Ptac* promoter, l*acI^q^*/expression vector	[72]
pT3sVamy	pSA10 derivative/expression of T3sVamy	This work
pT3sVgfp	pSA10 derivative/expression of T3sVgfp	This work
pΔsVgfp	pSA10 derivative/expression of ΔsVgfp; lacks T3 signal (EspF_20_)	This work
pCX340	Tc^r^, pBR-ori; *Ptrc* promoter, ‘*blaM* (TEM-1)/ vector for β-lactamase fusions	[26]
pT3s-Bla	pCX340 derivative/expression of T3s-Bla fusion	This work
pT3sVgfp-Bla	pCX340 derivative/expression of T3sVgfp-Bla fusion	This work
pT3sVamy-Bla	pCX340 derivative/expression of T3sVamy-Bla fusion	This work
pEGFP-N1	Km^r^, pUC/pSV40-ori, P_CMV_ promoter, enhanced GFP	Clontech
pCS2+MT	Ap^r^, pUC-ori, P_CMV_ promoter, 6xmyc-tag, vector	[74]

We next evaluated expression and secretion of the T3sV_HH_s by Western blot (WB). The anti-E-tag mAb detected the T3sV_HH_s (ca. 21–23 kDa) in the culture supernatants of wt EPEC but not in the Δ*escN* strain containing pT3sVamy and pT3sVgfp (Fig. 1C; top panel; lanes 1–6). In contrast, T3sV_HH_s were detected in the bacterial lysates of both the wt and Δ*escN* strains with these plasmids (Fig. 1C; lanes 7–12). The membranes were also probed with anti-GroEL mAb (Fig. 1C; bottom panels) to control for non-specific release of cytoplasmic EPEC proteins. The result showed no reactivity with anti-GroEL in culture supernatants (lanes 1–6) whereas strong signals were detected in bacterial lysates (lanes 7–12). Secretion of the T3sV_HH_s to culture supernatants was also observed in EHEC wt strain but not in the EHEC Δ*escN* strain (Fig. S2; Table 1). As an additional proof of the T3-dependent secretion, a derivative of pT3sVgfp was constructed in which the T3s was deleted (pΔsVgfp). WB of induced wt EPEC and EHEC carrying pΔsVgfp resulted in intracellular accumulation of ΔsVgfp but not in its secretion (Fig. S3). To determine whether secreted T3sV_HH_s remained as soluble proteins, and to rule out their aggregation or association with outer membrane vesicles (OMVs), the culture supernatants from induced EPEC were ultracentrifuged at 100,000 g. The presence of T3sV_HH_s in the soluble and pellet fractions was analyzed by WB (Fig. S4) showing that ≥95% of T3sV_HH_s are found as soluble proteins.

Taking advantage of the His-tag present at their C-termini, both T3sV_HH_s were purified (Fig. S5) with standard yields between 0.5–1 mg/L of culture supernatant. Binding activities of the purified T3sV_HH_s were tested by ELISA using antigens Amy, GFP or BSA (as negative control). Bound T3sV_HH_s were developed with anti-E-tag mAb demonstrating the specific antigen-binding activity of the T3sV_HH_s over a range of concentrations (Fig. 1D).

### Translocation of sdAbs into human cells

**Figure 2:**
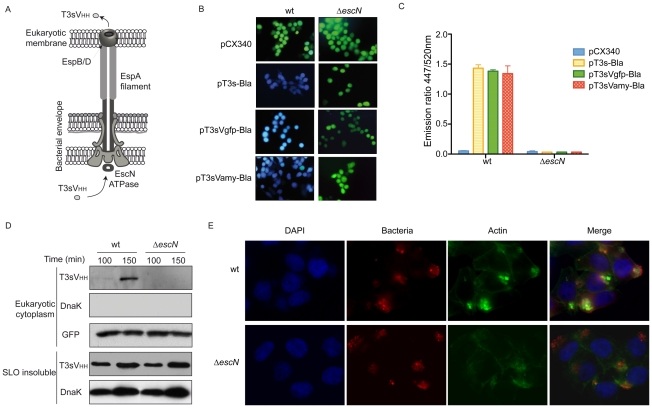
Translocation of sdAbs into HeLa cells using T3SS of EPEC. (**A**) Schematic representation of T3SS-complex from EPEC with EspB/D pore complex assembled in the mammalian cell plasma membrane. Injection of T3sNbs from the cytoplasm of the bacteria to the cytoplasm of the mammalian cell is shown. (**B**) Fluorescence microscopy images of cultured HeLa cells infected with EPEC wt (left column) and Δ*escN* (right column) strains, harbouring pCX340, pT3s-Bla, pT3sVamy-Bla or pT3sVgfp-Bla, as indicated, and incubated with CCF2/AM. Hydrolysis of CCF2 by translocated Bla changes the fluorescence emission of the cytosol of HeLa cells from 520 nm (green) to 447 nm (blue). (**C**) Quantification of the activity of translocated Bla by measuring the ratio of fluorescence at 447/520 nm in HeLa cells infected with the indicated bacteria. (**D**) Western blot of protein extracts after SLO treatment of HeLa cell cultures infected for the indicated time (min) with wt EPEC and Δ*escN* strains harboring pT3sVgfp. Eukaryotic cytoplasm extracts (top panels) were developed with anti-E-tag mAb to detect T3sV_HH_s, anti-DnaK mAb to control the absence of bacterial contamination, and anti-GFP mAb to test the efficacy of SLO pore formation in all samples and as a loading control. SLO-insoluble protein extracts (bottom panels), corresponding to EPEC bacteria and HeLa cell debris, were developed with anti-E-tag mAb, to show the expression level of T3sNb in bacteria, and with anti-DnaK mAb to control similar attachment of both strains to HeLa cells. (**E**) Immunofluorescence microscopy images of Hela cells infected with EPEC wt and Δ*escN* strains to demonstrate similar adhesion and microcolony formation of both strains in HeLa cells. Bacteria are labeled with anti-O127 serum (red), F-actin labeled with conjugated phalloidin (green), and DNA and cell nuclei labeled with DAPI (blue). Actin accumulation is only observed underneath wt EPEC.

We tested whether T3sV_HH_s can be injected into human cells with EPEC strain (Fig. 2A). Toward this end, vector pCX340 (Table 2), which encodes the TEM-1 β-lactamase (Bla) reporter devoid of its natural N-terminal Sec-dependent signal peptide [26], was used to generate T3s-Bla, T3sVamy-Bla, and T3sVgfp-Bla fusions (Fig. S6A). Plasmids pT3s-Bla and pCX340 were used as positive and negative controls of translocation, respectively. Cultured HeLa cells, infected with wt and Δ*escN* EPEC carrying these Bla-expressing plasmids, were incubated with the nonfluorescent esterified CCF2/AM substrate. Upon passive entry into the eukaryotic cell, CCF2/AM is transformed by eukaryotic esterases to the fluorescent substrate of Bla CCF2, which is mostly contained inside the eukaryotic cell [26]. Injection of Bla into the cytoplasm of the eukaryotic cells leads to hydrolysis of CCF2 changing its fluorescence emission from 520 nm (green) to 447 nm (blue), which could be detected under the fluorescence microscope or quantified in a fluorimeter. Examination by fluorescence microscopy revealed a clear shift to blue fluorescence of HeLa cells infected with wt EPEC carrying pT3s-Bla, pT3sVamy-Bla, or pT3sVgfp-Bla (Fig. 2B). In contrast, green fluorescence was observed in HeLa cells infected either with wt EPEC/pCX340 or with any of the Δ*escN* strains (Fig. 2B), demonstrating that the hydrolysis of CCF2 is only due to the Bla translocated into the cytosol of the HeLa cells. The fluorescence intensity of infected cells was also quantified in a fluorimeter and is shown as the ratio between blue emission fluorescence (447 nm) and green emission fluorescence (520 nm) (Fig. 2C). Expression of Bla fusions in wt and Δ*escN* EPEC was confirmed by WB with anti-Bla antibodies (Fig. S6B). Thus, these results demonstrate that EPEC carrying a functional T3SS are able to inject T3sV_HH_s-Bla fusions into human cells.

Next, we investigated whether T3sV_HH_s could be detected in the cytoplasm of infected HeLa cells. Toward this end we employed a fractionation method of the infected cells based on Streptolysin-O (SLO), a pore-forming cytolysin from *Streptococcus pyogenes* that selectively bind to cholesterol groups in the eukaryotic plasma membrane [32], [33]. HeLa cells were infected for 90 or 150 min with EPEC expressing T3sV_HH_s in the presence of IPTG, placed on ice and the monolayer washed with PBS. Following SLO treatment the cytoplasmic content of HeLa cells was collected (“Eukaryotic cytoplasm” extract). Ghost HeLa cells and bound bacteria were lysed in a SDS-containing buffer (“SLO-insoluble” extract). Protein extracts were subjected to WB revealing a time-dependent accumulation of T3sVgfp in the cytoplasm of HeLa cells infected with wt EPEC but not with EPEC Δ*escN* (T3sV_HH_ top panel, Fig. 2D). No sign of bacterial contamination was detected in the eukaryotic cytoplasmic extracts using a mAb directed against DnaK (Fig. 2D). Efficiency of the SLO-treatment in the different samples was controlled by WB developed with anti-GFP mAb (Fig. 2D). In addition, WB of the “SLO-insoluble” extracts demonstrated similar expression levels of T3sVgfp in wt EPEC and Δ*escN* strains (T3sV_HH_ bottom panel, Fig. 2D). The comparable signal from the “SLO-insoluble” extracts probed with anti-DnaK indicated similar cell attachment levels of wt EPEC and Δ*escN* (Fig. 2D). This was confirmed by fluorescence microscopy of the infected cultures (Fig. 2E), in which microcolonies of wt EPEC and Δ*escN* bacteria (red) were stained adhered to HeLa cells at similar levels, although actin pedestals are only observed in cells infected by wt EPEC (Fig. 2E, staining of F-actin in green).

Since wt EPEC also secretes T3sVgfp to the extracellular medium, we wanted to exclude the possibility that the T3sVgfp molecules detected in the cytoplasmic extracts entered the cells indirectly from the extracellular medium through the pores formed in the plasma membrane by the EspBD translocation pore or during the SLO-treatment (despite removal of the extracellular medium before addition of SLO). For this, HeLa cells were infected with wt EPEC harboring empty pSA10 (expressing a functional T3SS but not T3sVgfp) or EPEC Δ*escN*/pT3sVgfp (lacking a functional T3SS and thus unable to inject T3sVgfp). After 120 min of infection, the culture media was replaced by conditional, bacteria-free, medium obtained from an induced wt EPEC/pT3sVgfp culture (containing secreted T3sVgfp). After a further 30 min incubation, infected cells were fractionated with SLO into “eukaryotic cytoplasm” and “SLO-insoluble” extracts, as above. WB showed that T3sVgfp was not found in the “eukaryotic cytoplasm” (Fig. S7), hence ruling out the possibility that T3sVgfp molecules were entering HeLa cells from the extracellular medium. Taken together, the experiments with Bla reporter and biochemical fractions demonstrated that wt EPEC are able to directly inject T3sV_HH_s into human cells.

**Figure 3:**
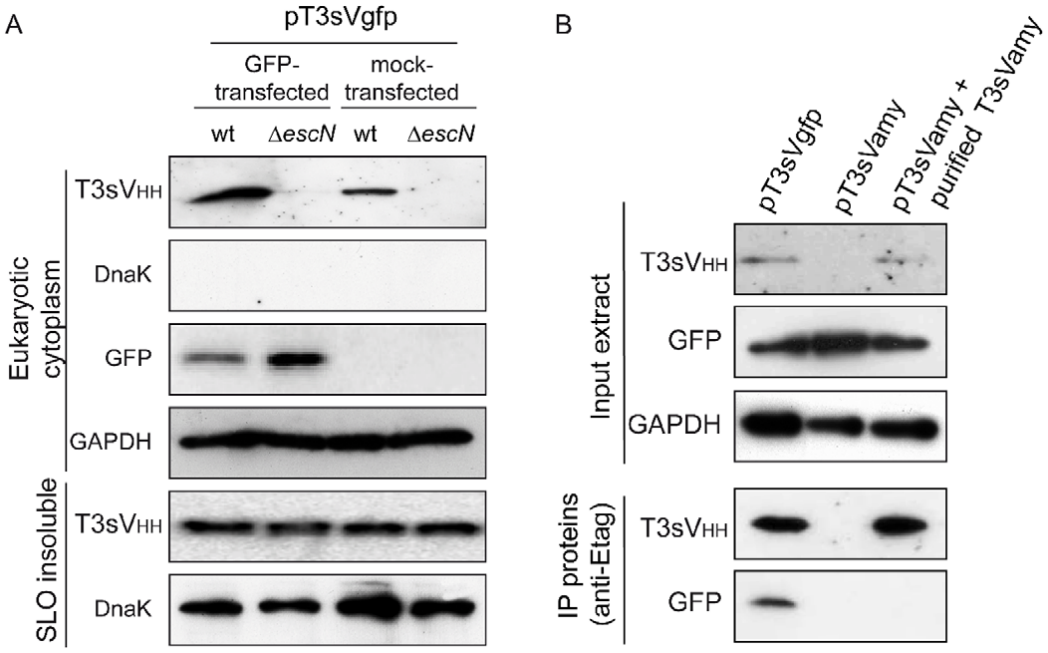
Antigen binding recognition by translocated sdAbs. (**A**) Antigen expression (GFP) increases the level of T3sVgfp detected in the cytoplasm of HeLa cells. Western blot of “eukaryotic cytoplasm” (top panels) and “SLO-insoluble” (bottom panels) protein extracts from infected HeLa cell cultures transfected with the indicated plasmids: pEGFPN1 (GFP) or control pCS2+MT (mock). Infections were carried out for 150 min with EPEC wt and Δ*escN* strains harboring pT3sVgfp. The level of T3sVgfp in the cytoplasmic extracts of HeLa cells was developed with anti-E-tag mAb (T3sV_HH_ top panel). Absence of bacterial contamination in eukaryotic cytoplasm protein extracts was controlled with anti-DnaK mAb (DnaK top panel). Transfection and expression of GFP was controlled with and anti-GFP mAb (GFP panel). Levels of human cytoplasmic GAPDH were determined with anti-GAPDH mAb to control equal SLO pore formation in all samples and as a loading control. SLO-insoluble protein extracts (bottom panels) were developed with anti-E-tag mAb to show the level of T3sVgfp in bacteria and with anti-DnaK mAb to control bacterial attachment. (**B**) Immunoprecipitation of T3sVgfp:GFP complexes from the cytoplasm of infected HeLa cells. Western blot of input eukaryotic cytoplasm protein extracts and immunoprecipitated proteins with anti-E-tag mAb bound to protein G-Sepharose resin. Input extracts were obtained by SLO treatment from pEGFP-N1-transfected HeLa cell cultures infected with wt EPEC strain carrying pT3sVgfp or pT3sVamy. Purified T3sVamy was added to one aliquot of input extract from cells infected with EPEC/pT3sVamy (lane 3) to reach a level similar to the translocated T3sVgfp (lane 1). Input extracts were developed with anti-E-tag, anti-GFP, and anti-GAPDH mAbs (top panels). Immunoprecipitated (IP) proteins were developed with anti-E-tag and anti-GFP mAbs (bottom panels).

We also investigated whether the presence of their cognate antigen influence the stability of T3sV_HH_s. Toward this end HeLa cells, transfected with pEGFP-N1 or with the empty vector pCS2+MT (Table 2), were infected with wt EPEC and EPEC Δ*escN* harboring pT3sVgfp. “Eukaryotic cytoplasm” and “SLO-insoluble” extracts were analyzed by WB revealing that the amount of T3sVgfp found in the cytoplasm of HeLa cells was ∼3-fold higher when GFP was expressed (Fig. 3A, T3sV_HH_ panel). Detection of the cytoplasmic enzyme glyceraldehyde 3-phosphate dehydrogenase (GAPDH) was used as a loading control of the “eukaryotic cytoplasm” extracts (Fig. 3A, GAPDH panel). WB with anti-DnaK demonstrated the absence of bacterial contamination in the “eukaryotic cytoplasm” extracts and similar signals in the “SLO-insoluble” extracts (Fig. 3A, DnaK panels). Therefore, higher levels of injected T3sV_HH_s are found when HeLa cells express the relevant antigen. We estimated that after 150 min infection in the presence of IPTG, an average of ∼7×10^5^ molecules of T3sVgfp per cell are found in pEGFPN1-transfected cells. This estimation was done by densitometry of the WB signals of T3sVgfp in “eukaryotic cytoplasm” extracts from three independent infections with the signals generated with known protein concentrations of purified T3sVgfp used as standard curve (Fig. S8).

### Antigen binding activity of injected sdAbs

To investigate formation of intracellular antigen-V_HH_ complexes, “eukaryotic cytoplasm” extracts from pEGFP-N1-transfected cells, infected with wt EPEC carrying pT3sVgfp or pT3sVamy, were immunoprecipitated (IP) with anti-E-tag mAb bound to protein G-beads. WB of the input extracts and IP proteins (Fig. 3B) revealed that GFP was specifically co-IP from the cytoplasmic extracts of cells infected with EPEC/pT3sVgfp demonstrating the formation of intracellular GFP-T3sVgfp complexes. Since T3sVamy does not accumulate at detectable levels in the cytoplasm of HeLa cells (likely caused by the absence of its antigen in the cytoplasm; see Discussion), purified T3sVamy was added to the input extract of cells infected with EPEC/pT3sVamy to provide a control of the specific co-IP of GFP with T3sVgfp (Fig. 3B). Similar amounts of GFP and GAPDH proteins were detected in the input extracts (Fig. 3B). These data provide direct evidence that the injected T3sV_HH_s have the capacity to bind their specific antigen in the cytoplasm of human cells.

### Translocation of sdAbs by attenuated EPEC

**Figure 4:**
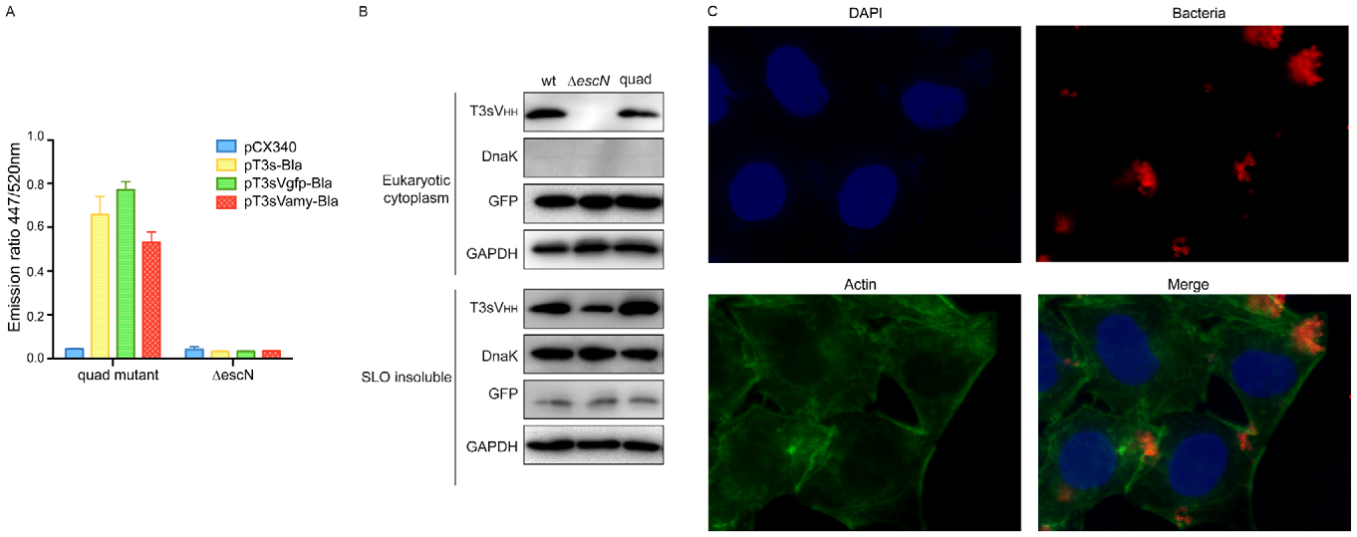
Injection of sdAbs by attenuated bacterial strain. (**A**) Quantification of the Bla activity by measuring the ratio of fluorescence at 447/520 nm in HeLa cells infected with quad mutant and Δ*escN* strains, carrying the indicated plasmids. (**B**) Western blot of “eukaryotic cytoplasm” (top panels) and “SLO-insoluble” (bottom panels) extracts from infected HeLa cell cultures with wt EPEC, quad mutant, and Δ*escN* strains harboring pT3sVgfp. WB developed as in Figs 2 and 3. (**C**) Immunofluorescence microscopy images of Hela cells infected with the quad mutant and Δ*escN* strains, to demonstrate the absence of actin pedestals and similar adhesion and microcolony formation by the attenuated quad mutant. Bacteria are labeled with anti-O127 serum (red), F-actin labeled with conjugated phalloidin (green), and DNA and cell nuclei labeled with DAPI (blue).

The EPEC wt strain is a pathogen causing strong cytopathic effects due to the injection of its natural repertoire of T3 effectors [14], [18]. Therefore most biotechnological applications will require using attenuated strains deficient in the major or all of the T3 effectors. To obtain a proof-of-principle of the injection of V_HH_s with attenuated bacteria, we employed quadruple (“quad”) mutant strain of EPEC that assembles functional injectisomes but carries four deletions in genes encoding the adhesin intimin and the effectors Tir, EspF and Map [34]. Bla translocation assays in HeLa cells infected with the “quad” mutant or with EPEC Δ*escN* (as a control) demonstrated the injection of T3s-Bla, T3sVamy-Bla and T3sVgfp-Bla by the *“*quad” mutant strain (Fig. 4A). The emission ratio at 447/550 nm obtained with the “quad” mutant were ∼60% those reached with wt EPEC (compare Fig. 2C and Fig.4A) suggesting a lower infection efficacy with the attenuated mutant. T3sVgfp was also detected in the cytoplasmic extracts of HeLa cells transfected with pEGFPN1 and infected with “quad” mutant” (Fig. 4B). Infection with wt EPEC and Δ*escN* bacteria, both carrying pT3sVgfp, were used as positive and negative controls, respectively. The level of translocated T3sVgfp by the “quad mutant” strain was found to be ∼60% that reached by the wt EPEC strain (Fig 4B, T3sV_HH_ top panel). Absence of bacterial contamination in the cytoplasmic extracts was confirmed with anti-DnaK and detection of GAPDH and GFP was used as a control of gel loading and of the efficacy of the SLO-treatment. “SLO-insoluble” extracts showed similar levels of T3sVgfp and DnaK in infections with EPEC wt and “quad” strains (Fig. 4B). Fluorescence microscopy of infected cells showed microcolonies of the “quad” strain on the surface of HeLa cells and the absence of F-actin pedestals (Fig. 4C). Altogether these data demonstrate an attenuated *E. coli* strain with a functional T3SS is capable of translocation of T3sV_HH_s into HeLa cells.

## Discussion

Expression of Ab fragments in the cytoplasm of human cells allows targeting of intracellular proteins that participate in disease and infection processes [7]. In this work we have shown that non-invasive *E. coli* bacteria carrying a functional T3SS are able to secrete and translocate to the cytoplasm of human cells sdAb fragments with full capacity to bind their cognate antigens. Single-domain V_HH_s appeared specially suited for this application given their potential as enzyme inhibitors, monomeric nature, stability, and size (2–3 nm diameter) [5] that fit in the protein channels of T3 needles and EspA filament [19], [35]. We have shown that T3-secreted V_HH_s present in the extracellular medium do not enter in the cytoplasm of HeLa cells via EspBD pores, and that translocation only occurs directly from bacteria to the mammalian cell. In addition, we obtained evidence of the formation of antigen-V_HH_ complexes in the cytoplasm of the infected cells and quantified that ∼7×10^5^ V_HH_ molecules accumulated per cell in the presence of antigen. Interestingly, we found that expression of the antigen in the cytoplasm of the mammalian cell increases the level of V_HH_s in the cytoplasm, which suggests that formation of antigen-V_HH_ complex could stabilize the intracellular V_HH_. Although the actual reason of this stabilization is unclear, it is possible that the higher molecular weight of the antigen-V_HH_ complex might reduce its susceptibility to proteolyic degradation *in vivo*. In this regard, detection of translocated T3sV_HH_-Bla in cells that do not express their cognate antigens could also be explained by their higher molecular weight of these fusions.

To establish this proof-of-principle we took advantage of non-invasive intestinal pathogenic *E. coli* strains that harbor a T3SS, such as EPEC and EHEC [12], [13]. Although *in vivo* infection by these strains is restricted to humans and certain animals (e.g. cattle), *in vitro* they can infect many mammalian cell lines from distinct cell types and species, including human and murine epithelial cells, fibroblasts, and macrophages among others [36]–[38]. Interestingly, the mouse pathogen *Citrobacter rodentium* also carries a *LEE* pathogenicity island encoding a T3SS almost identical to that found in EPEC and EHEC strains [39].

Applications of intrabody delivery by bacterial injection require the use non-pathogenic bacterial strains. In this study we employed an attenuated strain lacking the adhesin Intimin and three major T3-effectors (EspF, Map, Tir) of EPEC. In the absence of intimin and Tir EPEC looses its intimate adhesion and actin pedestal formation capabilities, while the lack of EspF and Map diminishes major cytopathic effects due to disruption of the mitochondria and activation of Rho GTPases [14], [34]. A study with human volunteers who ingested an EPEC null mutant in the intimin gene (*eae*) demonstrated a strong attenuation of this strain [40]. The EPEC strain E2348/69 used in this study encodes 21 effectors of the T3SS [12]. Accordingly, it would be possible to engineer an attenuated *E. coli* strain lacking all these effectors following a genome minimization approach [41]. Such bacterial strain will be extremely useful for antibody injection into mammalian cells. Another appealing strategy for bacterial injection of sdAbs is the use of commensal *E. coli* strains endowed with a functional T3SS. It has been reported that *E. coli* K-12 strain carrying the complete *LEE* from EPEC on a cosmid is able to induce actin polymerization in human cells *in vitro* [42]. However, the *LEE* is expressed weakly in *E. coli* K-12 [43] and, therefore, additional engineering is needed for efficient injection from *E. coli* K-12.

The use of extracellular bacteria with a T3SS as vectors for delivery of proteins (including intrabodies) into mammalian cells differs from other approaches that need the transfer of the protein-encoding gene by viral infection or transfection [8]. Invasive *E. coli* expressing the Invasin (Inv) from *Yersinia* and the Listeriolysin O (LLO) from *Listeria* have been employed previously to deliver proteins, DNA, and interfering shRNA into mammalian cells [11]. Upon cell invasion, invasive *E. coli* cells (Inv+ LLO+) are lysed in the phagosomes releasing their total cellular content. In contrast to this situation, non-invasive *E. coli* cells carrying a T3SS remain extracellular and could inject specifically the desired sdAb. In addition, extracellular *E. coli* bacteria are more sensitive to antibiotic treatment (e.g. gentamycin) facilitating their elimination from *in vitro* cultures as well as from whole animals *in vivo*.

The use of live bacteria has a great potential for delivery of therapeutic proteins *in vivo*, in specific organs or tissues of the animal, where they can produce a continuous supply of the polypeptide. For instance, probiotic strains of lactic acid bacteria have been used *in vivo* for the extracellular secretion of cytokines, enzymes, antibody fragments, etc. against infectious and inflammatory diseases [44]–[46]. Also mucosal and systemic infections with live attenuated invasive bacterial strains (e.g. *Salmonella, Listeria*) have been employed for intracellular delivery of antigens for vaccination and cytotoxins for tumor therapy [47]–[54]. Interestingly, probiotic *E. coli* strains are currently used as therapeutic agents in humans. For instance, colonization of the gastrointestinal tract by *E. coli* Nissle 1917 [55] is used to treat some inflammatory bowel diseases such as Crohn's disease [56], [57]. In addition, deliberate colonization of the urinary bladder with probiotic *E. coli* strain ABU83972, isolated from an asymptomatic bacteriuria patient [58], is being used to treat recurrent urinary tract infections by uropathogenic *E. coli* strains [59], [60]. Therefore, an attractive possibility is to engineer probiotic *E. coli* strains to carry a functional T3SS to deliver intracellularly therapeutic sdAbs targeting proteins involved in diseases such as inflammation and cancer in the gastrointestinal and urinary tracts. Systemic infections to treat other organs and solid tumors are not excluded [61], [62]. Importantly, sdAbs interfering the function of relevant intracellular targets involved in cell proliferation (e.g. Ras), apoptosis (e.g. Caspase-3), cell migration (e.g. Gelsolin), and HIV-replication proteins (e.g. Rev) have already been selected [63]–[66]. The levels of intracellular sdAbs reported here with the *E. coli* T3SS (10^5^–10^6^ molecules per cell) seem appropriated to modulate the activity of regulatory and cell-signaling proteins, which often have intracellular levels below 10^6^ molecules per cell [67], [68] and, in addition, could trigger a downstream signaling cascade that would amplify the initial effect of the intrabody.

In conclusion, we believe that injection of sdAbs into mammalian cells using non-pathogenic bacterial strains carrying a T3SS is a promising technology for *in vitro* and *in vivo* intrabody applications that target host cell functions and signaling pathways.

## Materials and Methods

### Bacterial strains, growth, induction and infection conditions

*Escherichia coli* strains used in this work are listed in Table 1 [12], [13], [34], [37], [69], [70]. Bacteria were grown at 37°C in Luria-Bertani (LB) agar plates, in liquid LB medium or in Dubelcco's modified Eagle's medium (DMEM), as indicated. Media were supplemented with appropriated antibiotics for selection. Antibiotics were used at the following concentrations: Ampicillin (Ap) 150 µg/ml; Chloramphenicol (Cm) 30 µg/ml; Kanamycin (Km) 40 µg/ml; Tetracycline (Tc) 10 µg/ml. For secretion into the extracellular media of sdAbs, 5 ml cultures were grown overnight in LB at 37°C under static conditions. Next day, these cultures were used to inoculate 15 ml of DMEM (initial OD_600_∼0.05) in a capped Falcon tube (Beckton Dickinson) and incubation continued at 37°C with shaking until OD_600_∼0.4. At this point, 0.1 mM isopropyl-1-thio-b-D-galactoside (IPTG) was added for 4 h. For infection experiments, overnight LB cultures (as above) were used to inoculate 15 ml of DMEM (initial OD_600_∼0.1) and the cultures were incubated under static conditions at 37°C with 5% CO_2_ for 2 h, as a pre-activation step. Bacteria from these cultures were used for infection of HeLa cell cultures (∼10^5^ cells/well in 24-well tissue culture plates; Falcon) at a multiplicity of infection (MOI) 300∶1 (bacterial CFUs: HeLa cells) and 0.1 mM IPTG was added. Infection continued at 37°C with 5% CO_2_ for the indicated time (90–150 min) and were stopped on ice.

### Plasmids and DNA constructs

Plasmids employed in this study are summarized in Table 2. Standard methods of DNA manipulation were used [71]. All DNA constructs were sequenced using an automated DNA sequencer (Perkin Elmer). Oligonucleotides were synthesized by Sigma Genosys (Table 3). Plasmid pSA10 is a vector that contains *lacI^q^* repressor and a multiple cloning site under the control of *Ptac* promoter [72]. Plasmid pT3sVamy contains a DNA fragment of 549 bp, cloned at the *Eco*RI site of pSA10 under the P*tac* promoter, which encodes the V_HH_ anti-amylase (Vamy) fused to the T3-signal EspF_20_ at its 5′-end and a six-histidine (His) tag and the 12-amino acid epitope E-tag (GAPVPYPDPLEP) at its 3′-end. This DNA fragment was obtained by *Eco*RI digestion of a 560 bp DNA product generated by homology-driven PCR, fusing two PCR subfragments with a final amplification of the fused product with oligonucleotides R1-Xb-SD-espF y RI-stop-E (Table 3). Subfragment 1 (119 bp), containing EspF_20_ signal, was amplified from genomic DNA from EDL933*stx* with primers R1-Xb-SD-espF and SfiI-espF. Subfragment 2 (517 bp), corresponding to Vamy with His and E-tag epitopes, was amplified using plasmid pEHLYA4SDVamy as a template and oligonucleotides SfiIVamy and RI-stop-E as primers. Plasmid pEHLYA4SDVamy is a derivative of pEHLYA2SDVamy [28] with the His and the E-tag epitope at the 3′end of Vamy. Plasmid pT3sVgfp was obtained by *Sfi*I and *Not*I digestion of pT3sVamy, substituting Vamy coding sequence by Vgfp. The DNA encoding Vgfp was obtained by PCR using plasmid pcAbGFP4 [73] as template and oligonucleotides Vhh-sfiI2 and Vhh-NotI2 as primers. The amplified fragment was digested with *Sfi*I and *Not*I and the resulting 358 bp DNA molecule was cloned in the backbone of vector pT3sVamy (∼4.3 kb) digested with the same enzymes. Plasmid pΔsVgfp was constructed by amplication of a DNA segment, encoding Vgfp-His-E-tag devoid of the T3-signal, from pT3sVgfp template with oligonucleotides Δsign-EcoRI and RI-stop-E. The ∼0.5 kb PCR product was digested *Eco*RI and inserted in the same site of vector pSA10 under the control of *Ptac* promoter.

**Table 3:** Oligonucleotides used in this study.

Name	Sequence (*5*′*-3*′)
RI-Xb-SD-espF	CCGGAATTCTCTAGAAAGAGGCATAAATTATGCTTAATGGAATTAGTA
SfiI-espF	CTGCACCTGAGCCATGGCCGGCTGGGCCGCTGCGATACCTACAAGCTGCCGCCCTA
SfiIVamy	CTTGTAGGTATCGCAGCGGCCCAGCCGGCCATGGCTCAGGTGCAGCTG
RI-stop-E	CCGGAATTCTCATTAGGCCGGTTCCAGCGGATCCGGATACGGCAC
Vhh-SfiI2	GTCCTCGCAACTGCGGCCCAGCCGGCCATGGCTCAGGTGCAGCTGGTGGA
Vhh-NotI2	GGACTAGTGCGGCCGCTGAGGAGACGGTGACCTGGGT
NdeI-espF	CCGGATCCATATGCTTAATGGAATTAGTAACGCTGCTTCT
EcoRI-espF	GGTGCGAATTCGCTGCGATACCTACAAGCTGCCGCCCTA
EcoRI-TEM	GCGGCAGCTTGTAGGTATCGCAGCGAATTCGCACCCAGAAACGCTGGTGA
BamHI-tetra	ATGCGTCCGGCGTAGAGGATCCACAGGACGGGT
NdeI-espFVamy	GGGAATTCCATATGCTTAATGGAATTAGTAACGCTGCT
EcoRIVamy-espF	CCGGAATTCGCGGCCGGTTCCAGCGGATCCGGATA

Plasmids pT3s-Bla and pT3sVamy-Bla are derivatives of pCX340 [26], a vector employed to make fusions with the TEM β-lactamase lacking its Sec-dependent-signal peptide (‘*bla*M). To construct pT3s-Bla, first an 83 bp DNA segment encoding the T3-signal of EspF was amplified from genomic DNA from EDL933*stx* with the oligonucleotides NdeI-espF and EcoRI-espF. Next, this DNA segment was fused, by homology-driven PCR, with a 1.2 kb DNA fragment encoding (‘*bla*M), that had been amplified from pCX340 with oligonucleotides EcoRI-TEM and BamHI-tetra. The resulting 1.3 kb fragment, amplified with oligonucleotides NdeI-espF and BamHI-tetra, was digested with NdeI and BamHI and ligated with backbone fragment of pCX340 digested with the same enzymes. To construct pT3sVamy-Bla, a DNA fragment encoding T3sVamy was amplified from plasmid pT3sVamy with oligonucleotides NdeI-espFVamy and EcoRIVamy-espF, digested with *Nde*I and *Eco*RI and ligated in the same sites of pCX340.

### *In vitro* cell culture and plasmid transfection

The human epithelial cell line HeLa clone HtTA1 was grown as monolayer in DMEM, supplemented with 10% fetal bovine serum (FBS) and 2 mM glutamine, at 37°C with 5% CO_2_. For transfection, HeLa cells were seed in tissue 24-well culture plates (∼10^5^ cells/well), grown for 20 h at 37°C with 5% CO_2_. Plasmid pEGFPN1 (Clontech) or pCS2+MT [74] was added (0.6 µg DNA/well) to the cultures in calcium phosphate [75]. After 22 h incubation, the medium was removed, wells were washed three times with PBS, filled with 1 ml of complete medium and incubated for 1 h at 37°C with 5% CO_2_. This medium was replaced by serum-free medium before cell cultures were infected as described above.

### SDS-PAGE and Western blot analysis

Sodium Dodecy Sulfate–Polyacrylamide gel electrophoresis (SDS-PAGE) and Western blot was performed following standard methods [71] using the Miniprotean III system (Bio-Rad). Proteins separated by SDS-PAGE (in 10 or 12% gels) were stained with Coomassie Blue R-250 (Bio-Rad) or transferred to polyvinylidene difluoride membrane (PVDF, Immobilon-P, Millipore) as described previously [76]. Antibodies employed for Western blot were: anti-E-tag mAb conjugated to peroxidase (POD) (1∶5000; GE Amersham Biosciences); anti-E-tag mAb (Phadia; 0.5 mg/ml), anti-GroEL mAb-POD (1∶5000; Sigma); anti-β-lactamase mAb (1∶1000; QED Bioscience); anti-GFP mAb (1∶1000; Roche); anti-GAPDH (1∶2000; Santa Cruz Biotechnology); anti-DnaK (1∶5000; Stressgen). Unconjugated mAbs were detected with anti-mouse IgG-POD (1∶5000; Sigma) as secondary antibody. Membranes were developed by chemiluminiscence using Immun-Star WesternC kit (Bio-Rad) and exposed to X-ray films (Konica) and to a ChemiDoc XRS+ system for quantification (Bio-Rad).

### Analysis of secreted and cellular proteins in *E. coli* strains

Whole-cell protein extracts from induced *E. coli* cultures were obtained from cells harvested by centrifugation (3000×g, 5 min) from 1 ml aliquot of liquid cultures (OD_600_∼1.2), resuspended in 100 µl of phosphate-buffered saline (PBS), and mixed with the same volume of 2X SDS-PAGE sample buffer. Samples were boiled for 10 min, briefly sonicated (5 sec; Labsonic B Braun), and centrifuged (14000×g, 5 min) to remove insoluble material before loading on SDS-PAGE. For analysis of proteins secreted in the culture medium, culture supernatants obtained after centrifugation (3000×g, 15 min) were filtered utilizing a 0.22-µm sterile low-protein binding PVDF filter unit (Millex GV, Millipore) and the serine-protease inhibitor phenyl-methyl-sulphonyl-fluoride (PMSF) was added to 1 mM final concentration. The proteins in the filtered-culture supernatants were mixed with 1/5th of the volume of SDS-PAGE sample buffer (5X) for WB or were precipitated with trichloroacetic acid (TCA 20% w/v; Merck) for Coomassie staining. After centrifugation (14000×g, 15 min), TCA-precipitated protein pellets were rinsed with cold acetone (−20°C) and resuspended in SDS-PAGE sample buffer (1/10th of the precipitated volume). Purification of His-tagged T3-secreted sdAbs with Talon resin (Clontech) is described below. To test the solubility of the T3-secreted sdAbs, the filtered-culture supernantants (see above) were centrifuged at 100.000×g in a Beckman TL-100 ultracentrifuge for 1 h at 4°C. The supernatant (Soluble) and the pellet (insoluble) fractions obtained after this centrifugation were adjusted to the same final volume in SDS-PAGE sample buffer, boiled and analyzed by WB.

### Purification of T3-secreted sdAbs

Cultures of *E. coli* EPEC with the indicated plasmids (pT3sVamy or pT3sVgfp) were grown at 37°C with shaking in 200 ml of DMEM with ampicillin (using a capped bottle). IPTG was added at 0.1 mM final concentration when the OD_600_ reached ∼0.4 and incubation continued for 4 h. Culture supernatants were filtered and PMSF was added as described above. Next, supernatants were equilibrated to PBS 1X and incubated overnight at 4°C with 2 ml of Cobalt-containing chromatography resin (Talon, Clontech) for binding of His-tagged sdAbs. Resin was packed in a column, washed 4 times with 10 ml of PBS containing 5 mM imidazole, and eluted in 1 ml aliquots with PBS containing 100 mM imidazole. Eluted fractions were stored at 4°C.

### Enzyme-linked immunosorbent assays (ELISA)

ELISA conditions were based on those described previously [76]. Briefly, 96-well immunoplates (Maxisorp, Nunc) were coated for 2 h at room temperature with purified antigens (10 µg/ml) in PBS. Antigens employed: alpha-amylase (Amy; Sigma), the green fluorescent protein (GFP; Upstate), bovine serum albumin (BSA, Roche). Plates were washed with PBS and blocked in PBS buffer containing 3% (w/v) non-fat milk, before incubation with purified T3-secreted sdAbs or filtered-culture supernatants obtained after IPTG induction (at the indicated concentration or dilution in the same buffer). After PBS-wash, bound E-tagged sdAbs were revealed with anti-E-tag mAb-POD (1∶2000) and developed with *o-*phenylenediamine (OPD, Sigma) and H_2_O_2_ (Sigma). The OD at 490 nm of the plates was determined in a microplate reader (Bio-Rad).

### β-lactamase translocation assay

We followed the method described by [26]. Briefly, the indicated bacterial strains were used to infect HeLa cells grown *in vitro* in 8-well Falcon culture slides (Beckton Dickinson), IPTG was added for induction and incubation continued for 90 min. The medium was removed and cells were washed three times with Hank's balance salt solution (HBSS). Next, 200 µl of HBSS and 40 µl of the β-lactamase substrate CCF2/AM mix (K1024, Invitrogen) were added. Cells were incubated for additional 90 min at room temperature in the dark, washed three times with HBSS and analyzed by fluorescence microscopy (Nikon Eclipse E600, excitation UV light 330–380 nm). For quantitative analysis of Bla translocation, HeLa cells were seeded in a 96-well opaque plate (Nunc) at aprox. 85% confluence (∼2×10^4^ cells/well). After 16 h incubation at 37°C with 5% CO_2_, infection were done with pre-activated EPEC strains in serum-free DMEM and the cultures were further incubated for 30 min before addition of IPTG, and 60 min after this addition. Infections were washed three times with HBSS, and 200 µl/well of HBSS were added plus 20 µl of CCF2/AM substrate mix. Samples were incubated for 90 min in the dark, washed three times with HBSS and finally 100 µl/well of HBSS were added. Plates were read in a FLUOstar Optima Microplate Fluorometer with a filter set 450/520 nm.

### Streptolysin-O (SLO) fractionation of infected cell cultures

Conditions of SLO treatment were based on those described by [77] with some modifications. Briefly, 24-well plates containing infected HeLa cell cultures (as described above) were placed on ice and washed three times with 1 ml/well of freshly prepared cold SLO-buffer (150 mM sucrose, 25 mM Hepes pH 7.4, 150 mM K-acetate, 2.5 mM MgCl_2_, 4 mM EGTA, 2 mM DTT). Next, 175 µl/well of SLO-buffer containing 10 µg/ml of Streptolysin O (purchase from Prof. Sucharit Bhakdi's laboratory, Institute of Medical Microbiology and Hygiene, Hochhaus am Augustusplatz, Mainz, Germany) were added followed by 15 min of incubation on ice to allow binding of SLO to cells. After this incubation, unbound SLO was removed by washing three times with 1 ml/well of cold SLO buffer and 175 µl/well of this buffer was added. Plates were incubated at 37°C for 15 min for SLO-pore formation to allow release of the cytosolic content of HeLa cells (which was confirmed by observation in an inverted light microscope; Carl Zeiss). Extracellular media containing released cytoplasmic proteins were collected from plates. Protease inhibitors were added (Complete EDTA-free Protease Inhibitors Cocktail, Roche) to these extracts and centrifuged at 4500×g (15 min, 4°C) in order to eliminate any bacteria and cells detached from plates. This supernatant was collected and centrifuged at 16000×g (15 min, 4°C). The final resulting supernatant was referred to as “eukaryotic cytoplasm” protein extract. Eukaryotic cells debris and bacteria that remained attached to plates after SLO treatment were collected in 175 µl/well of a SDS-PAGE sample buffer (1x) and this extract was referred to as “SLO-insoluble” protein extract.

### Immunoprecipitation assays

Eukaryotic cytoplasm protein extracts (350 µl obtained as described above from two infected tissue culture wells) were incubated with 40 µl of anti-E-tag mAb bound to protein G-Sepharose resin. Anti-E-tag mAb (1 mg, Phadia) was previously crosslinked to protein G-Sepharose resin (1 ml, Sigma) with dimethyl pimelimidate dihydrochloride (DMP; Sigma). After 16 h incubation at 4°C in an orbital shaker, the resin was collected by centrifugation (800×g, 1 min) and washed three times with 1.5 ml of 200 mM sodium phosphate buffer (pH 8.2). Bound proteins were eluted from resin by incubation with 60 µl of 0.1 M glycine pH 2.8 (10 min at room temperature) followed by centrifugation (800×g, 1 min). Supernatants were collected and 30 µl of 200 mM sodium phosphate buffer (pH 8.2) was added for pH neutralization, referred to as immunoprecipitated (IP) proteins. Routinely, 12 µl of these IP proteins were mixed with 3 µl of 5X SDS-PAGE sample buffer, boiled and gel-loaded for Western blot analysis.

### Immunofluorescence microscopy

Infected HeLa cell cultures, grown on coverslips in 24-well plates, were washed three times with 1 ml/well of PBS, fixed with 3% (w/v) paraformaldehyde (in PBS) for 20 min at room temperature, and washed again with PBS three times. Cells were permeabilized by incubation with PBS containing 0.1% (v/v) of Triton X-100 (Sigma) for 5 min. To label EPEC, a rabbit polyclonal anti-O127 serum was diluted 1∶200 in PBS with 10% donkey serum (Jackson Immunoresearch) and incubated 1 h at room temperature. Coverslips were washed three times with PBS and goat anti-rabbit IgG-Texas Red conjugated secondary antibody (1∶500 in PBS with 10% donkey serum; Molecular Probes) was added along with Oregon-Green conjugated phalloidin (1∶100; Invitrogen) and DAPI (1∶1000; Sigma) to label F-actin and DNA, respectively. Coverslips were washed three times with PBS after incubation and 4 µl of mounting medium (DAKO) was added. Coverslips were analyzed by conventional epifluorescence microscopy using a Zeiss Axio imager microscope.

## Supporting Information

Figure S1 **Scheme of plasmid vectors used for expression of T3sV_HH_ fusions.** The sequence encoding the first 20 amino acids of EspF effector (T3s) is fused to the corresponding V_HH_ (Vamy or Vgfp in pT3sVamy or pT3sVgfp, respectively). Epitope tags (His and E-tag) at the C-termini of fusions and unique restriction sites SfiI, NcoI and NotI flanking V_HH_ domain are indicated. Gene constructs are under the control of the IPTG-inducible Ptac promoter. The presence of lacI^q^ repressor, transcriptional terminators (T1,T2) from 5S ribosomal RNA gene, ampicillin-resistance (amp^r^) gene, and origin of replication (ori) are also shown.
(TIF)

Figure S2 **Secretion of T3sV_HH_s by EHEC.** (**a**) Proteins found in extracellular media (Culture supernantants; lanes 1–6) and cells (Bacterial lysates; lanes 7–12) from cultures of wild type EHEC or Δ*escN* mutant strains carrying plasmids pSA10, pT3sVamy, or pT3sVgfp, as indicated, analyzed by Western blot with mAb anti-Etag (top panels), to detect T3sV_HH_ fusions, or with anti-GroEL (bottom panels) to control the absence of bacterial lysis. Cultures were grown at 37°C in DMEM and induced with 0.1 mM IPTG for 4 h. **(b)** SDS-PAGE and Coomassie staining of proteins found in the extracellular media of cultures of EHEC and Δ*escN* mutant strains carrying the indicated plasmids and induced as in (a). The protein bands of T3sV_HH_ fusions, T3SS- effectors EspA, EspB, EspD, and that of the Sec-dependent autotransporter EspP, are labelled. Size in kDa of protein standards for SDS-PAGE is shown on the left.
(TIF)

Figure S3 **Requirement of the T3-signal for secretion of V_HH_s**. Western blot with anti-E-tag mAb-POD of proteins found in extracellular media (Culture supernatants) and cells (Bacterial lysates) from cultures of wild type EPEC (top panels) and EHEC (bottom panels) carrying plasmids pT3sVgfp or pΔsVgfp (lacking the T3-signal) as indicated. Cultures were grown at 37°C in DMEM and induced with 0.1 mM IPTG for 4 h.
(TIF)

Figure S4 **Solubility of the T3-secreted V_HH_s**. Extracellular media from induced cultures of EPEC carrying plasmids pT3sVgfp or pT3sVamy, as indicated, were ultracentrifuged (100.000×g, 1 h) and proteins present in the resulting supernatants (S) and pellet (P) fractions were analyzed by Western blot with anti-E-tag mAb-POD.
(TIF)

Figure S5 **Purified T3sV_HH_s from culture supernatants.** Coomassie stained SDS-polyacrylamide gel of metal affinity purified His-tagged T3sVgfp and T3sVamy from extracellular media of EPEC strains harbouring pT3sVgfp or pT3sVamy. Size in kDa of protein standards for SDS-PAGE is shown on the left.
(TIF)

Figure S6 **Expression of β-lactamase fusions in EPEC. A)** Scheme of β-lactamase (Bla) gene fusions in plasmids pCX340, pT3s-Bla, pT3sVgfp-Bla and pT3sVamy-Bla. The position of Ptac promoter, T3 signal and V_HH_ sequence, are indicated. **B)** Western blot developed with anti-β-lactamase mAb of whole cells protein extracts from induced EPEC wild type and Δ*escN* strains carrying the indicated Bla plasmid. Size in kDa of protein standards for SDS-PAGE is shown on the right.
(TIF)

Figure S7 **T3sV_HH_s do not enter into HeLa cells from extracellular media.** Western blot of “eukaryotic cytoplasm” (top panels) and “SLO-insoluble” (bottom panels) protein extracts from infected HeLa cell cultures with EPEC wt/pSA10 and EPEC Δ*escN/*pT3sVgfp in which their extracellular media were replaced by medium containing T3sVgfp from induced EPEC wt/pT3sVgfp. Western blot of “eukaryotic cytoplasm” extracts are developed with anti-E-tag to detect T3sV_HH_, anti-DnaK to control the absence of bacterial contamination and anti-GFP mAb to test the efficacy of SLO pore formation. Western blot of “SLO insoluble” extracts are developed with anti-E-tag to show the expression of T3sV_HH_ in bacteria and anti-DnaK mAb to control attachment of both strains to HeLa cells.
(TIF)

Figure S8 **Quantification of T3sV_HH_s molecules injected.** “Eukaryotic cytoplasm” protein extracts of pEGFPN1-transfected HeLa cells, infected with EPEC wt/pT3sVgfp, were analyzed by Western blot and the chemiluminiscence intensity of the protein band corresponding to T3sV_HH_ determined by densitometry in a Chemi-Doc (Bio-Rad). The average intensity signal of T3sV_HH_ corresponding to ca. 2300 HeLa cells from three independent infection experiments (closed circle) is interpolated to a stardard curve generated with the intesity of protein bands from samples of purified T3sVgfp of known concentration (open circles).
(TIF)
