# Supplementary figures and images for: Direct Injection of Functional Single-Domain Antibodies from E. coli into Human Cells
Source: PLoS One. 2010 Dec 8;5(12):e15227. doi: 10.1371/journal.pone.0015227 (PMC2999559; doi:10.1371/journal.pone.0015227)

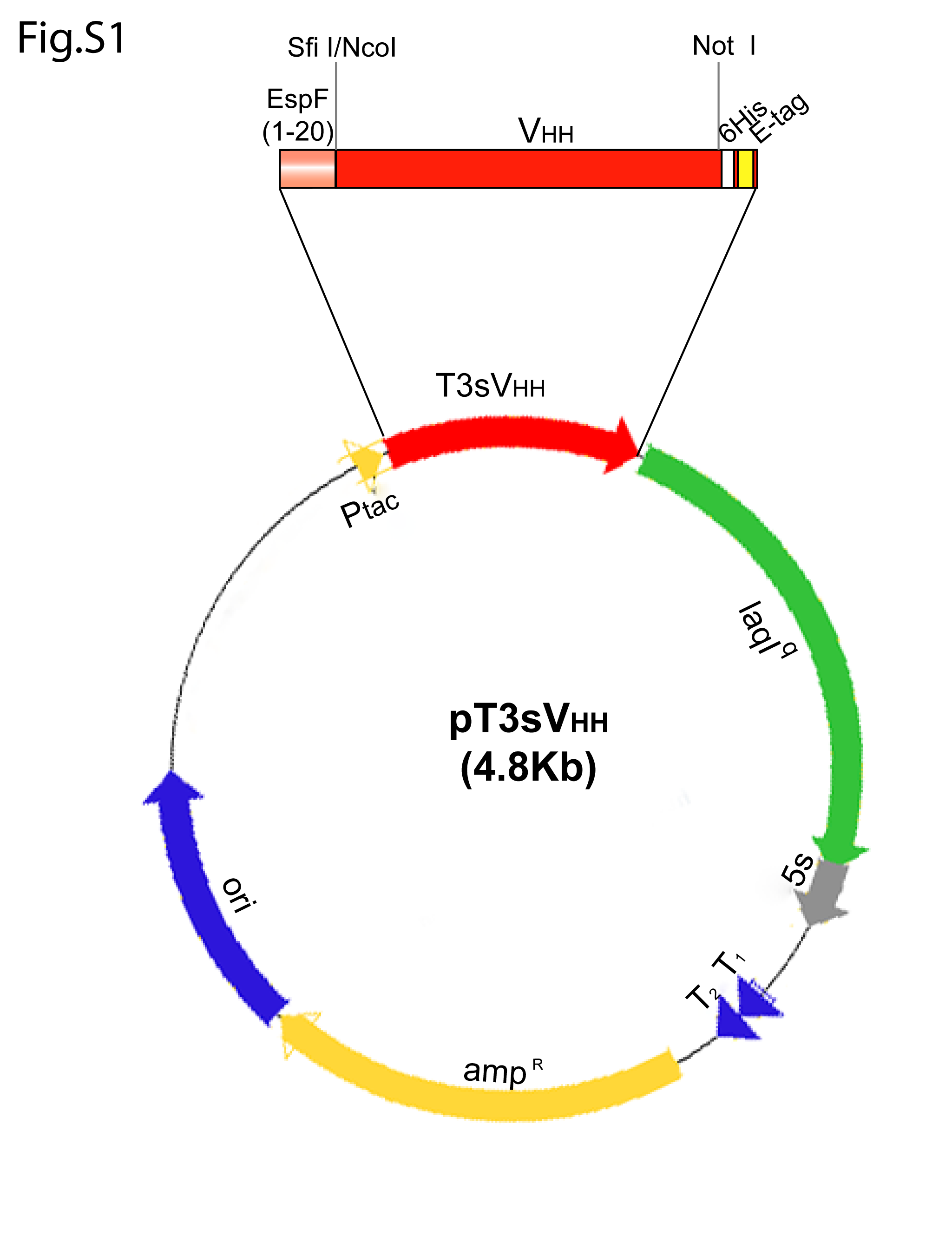

Supplement: Figure S1 — Scheme of plasmid vectors used for expression of T3sVHH fusions. The sequence encoding the first 20 amino acids of EspF effector (T3s) is fused to the corresponding VHH (Vamy or Vgfp in pT3sVamy or pT3sVgfp, respectively). Epitope tags (His and E-tag) at the C-termini of fusions and unique restriction sites SfiI, NcoI and NotI flanking VHH domain are indicated. Gene constructs are under the control of the IPTG-inducible Ptac promoter. The presence of lacIq repressor, transcriptional terminators (T1,T2) from 5S ribosomal RNA gene, ampicillin-resistance (ampr) gene, and origin of replication (ori) are also shown. (TIF) [file pone.0015227.s001.tif]

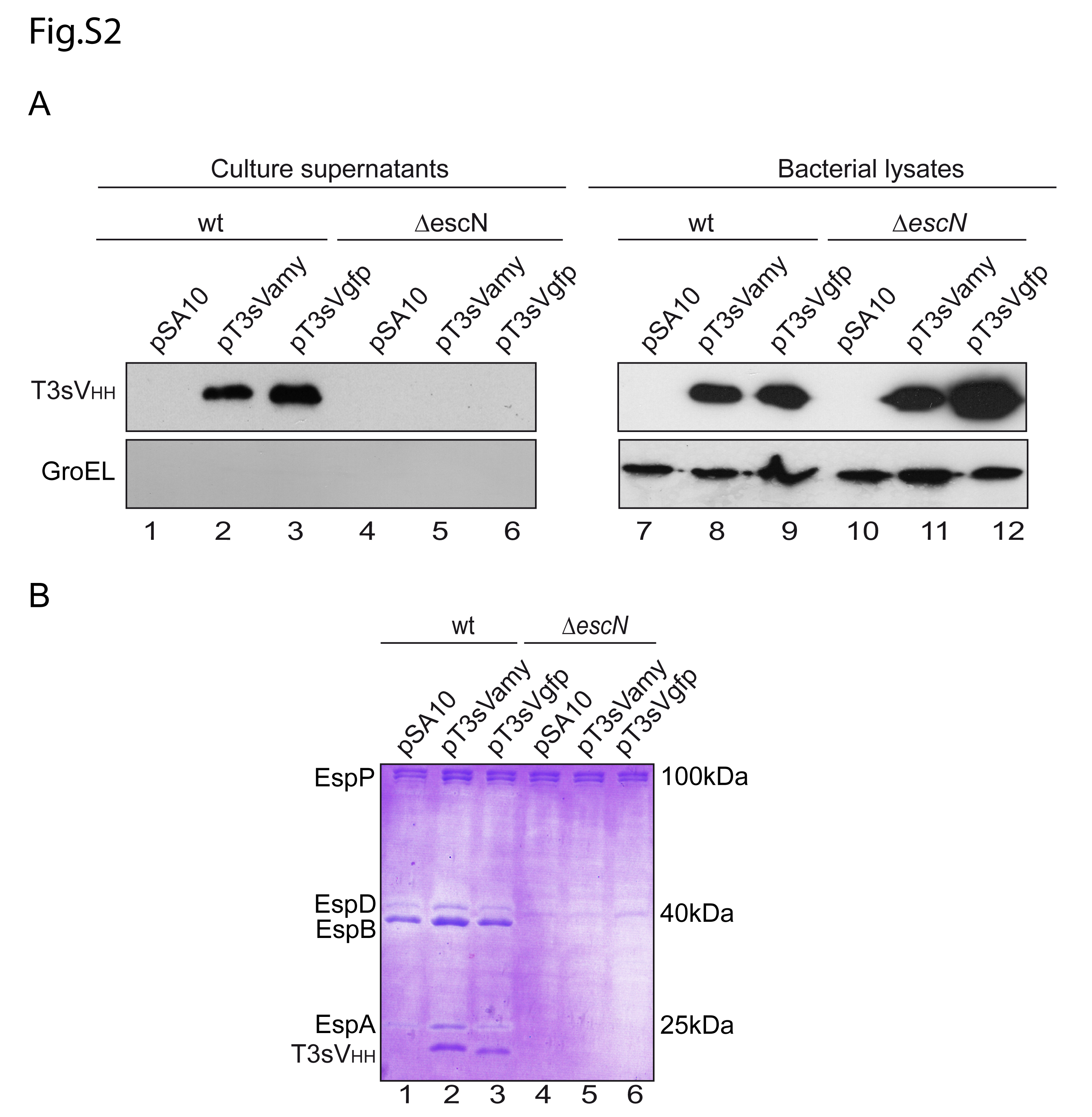

Supplement: Figure S2 — Secretion of T3sVHHs by EHEC. (a) Proteins found in extracellular media (Culture supernantants; lanes 1–6) and cells (Bacterial lysates; lanes 7–12) from cultures of wild type EHEC or ΔescN mutant strains carrying plasmids pSA10, pT3sVamy, or pT3sVgfp, as indicated, analyzed by Western blot with mAb anti-Etag (top panels), to detect T3sVHH fusions, or with anti-GroEL (bottom panels) to control the absence of bacterial lysis. Cultures were grown at 37°C in DMEM and induced with 0.1 mM IPTG for 4 h. (b) SDS-PAGE and Coomassie staining of proteins found in the extracellular media of cultures of EHEC and ΔescN mutant strains carrying the indicated plasmids and induced as in (a). The protein bands of T3sVHH fusions, T3SS- effectors EspA, EspB, EspD, and that of the Sec-dependent autotransporter EspP, are labelled. Size in kDa of protein standards for SDS-PAGE is shown on the left. (TIF) [file pone.0015227.s002.tif]

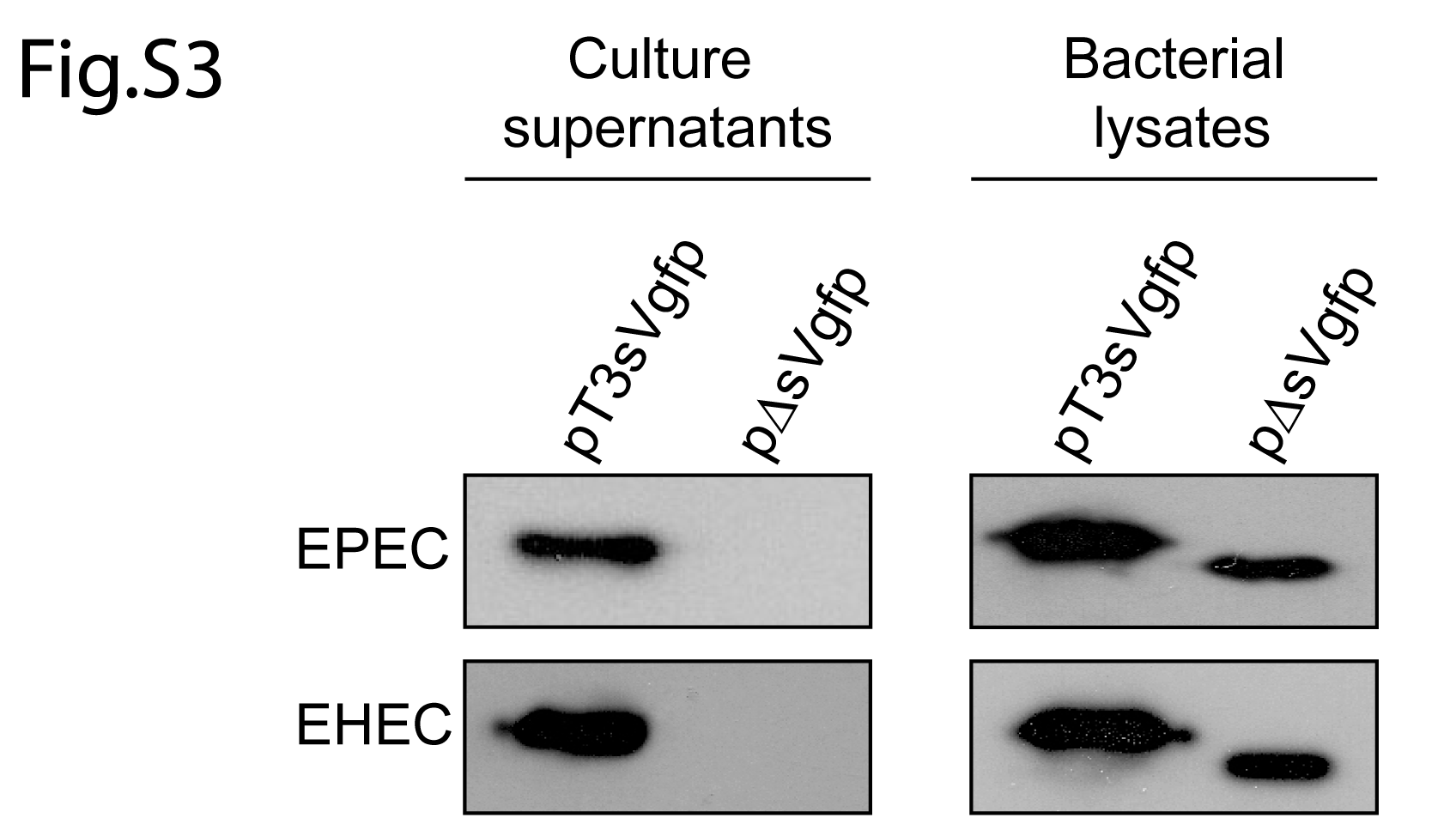

Supplement: Figure S3 — Requirement of the T3-signal for secretion of VHHs. Western blot with anti-E-tag mAb-POD of proteins found in extracellular media (Culture supernatants) and cells (Bacterial lysates) from cultures of wild type EPEC (top panels) and EHEC (bottom panels) carrying plasmids pT3sVgfp or pΔsVgfp (lacking the T3-signal) as indicated. Cultures were grown at 37°C in DMEM and induced with 0.1 mM IPTG for 4 h. (TIF) [file pone.0015227.s003.tif]

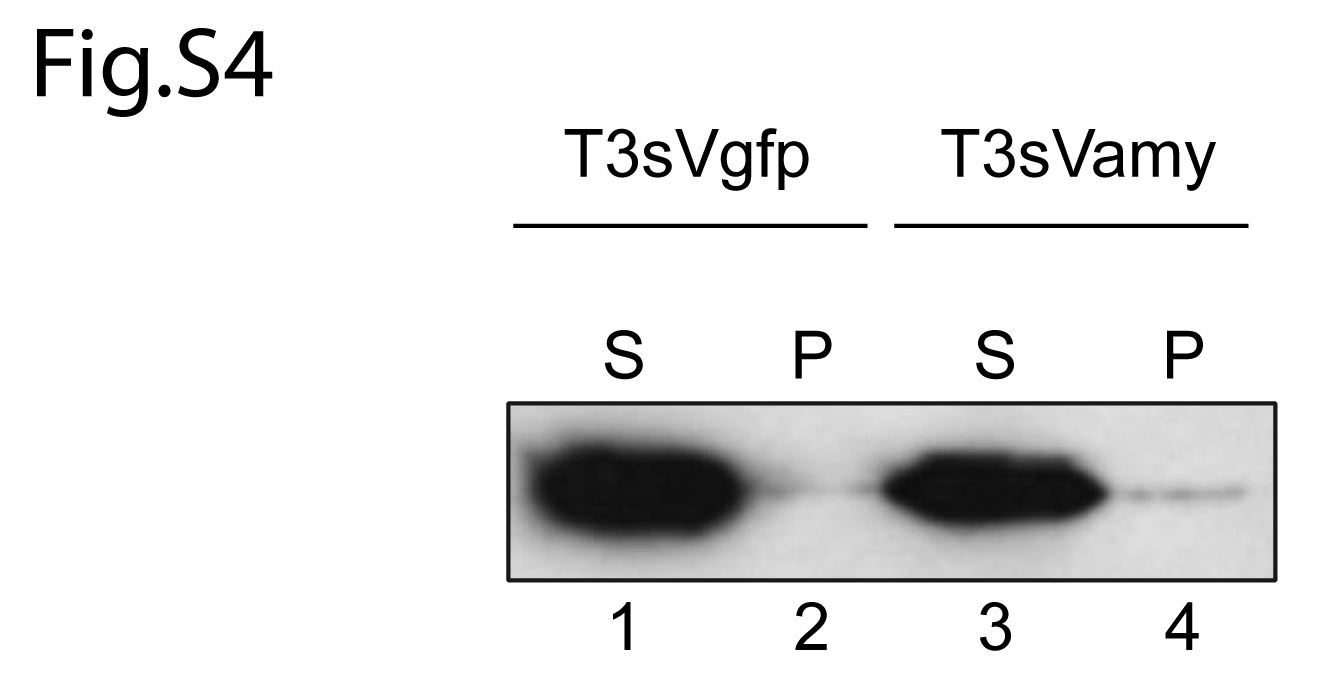

Supplement: Figure S4 — Solubility of the T3-secreted VHHs. Extracellular media from induced cultures of EPEC carrying plasmids pT3sVgfp or pT3sVamy, as indicated, were ultracentrifuged (100.000×g, 1 h) and proteins present in the resulting supernatants (S) and pellet (P) fractions were analyzed by Western blot with anti-E-tag mAb-POD. (TIF) [file pone.0015227.s004.tif]

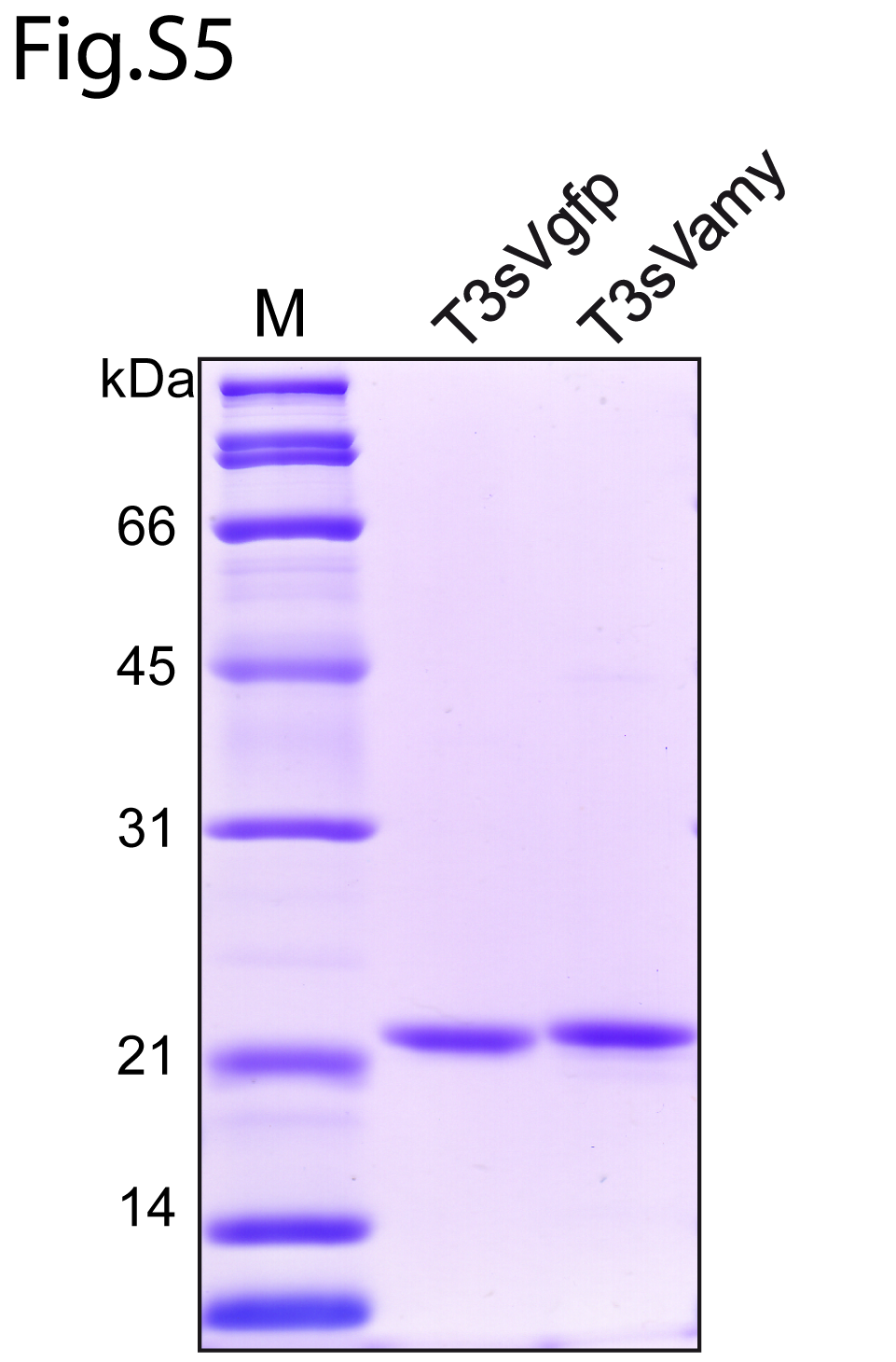

Supplement: Figure S5 — Purified T3sVHHs from culture supernatants. Coomassie stained SDS-polyacrylamide gel of metal affinity purified His-tagged T3sVgfp and T3sVamy from extracellular media of EPEC strains harbouring pT3sVgfp or pT3sVamy. Size in kDa of protein standards for SDS-PAGE is shown on the left. (TIF) [file pone.0015227.s005.tif]

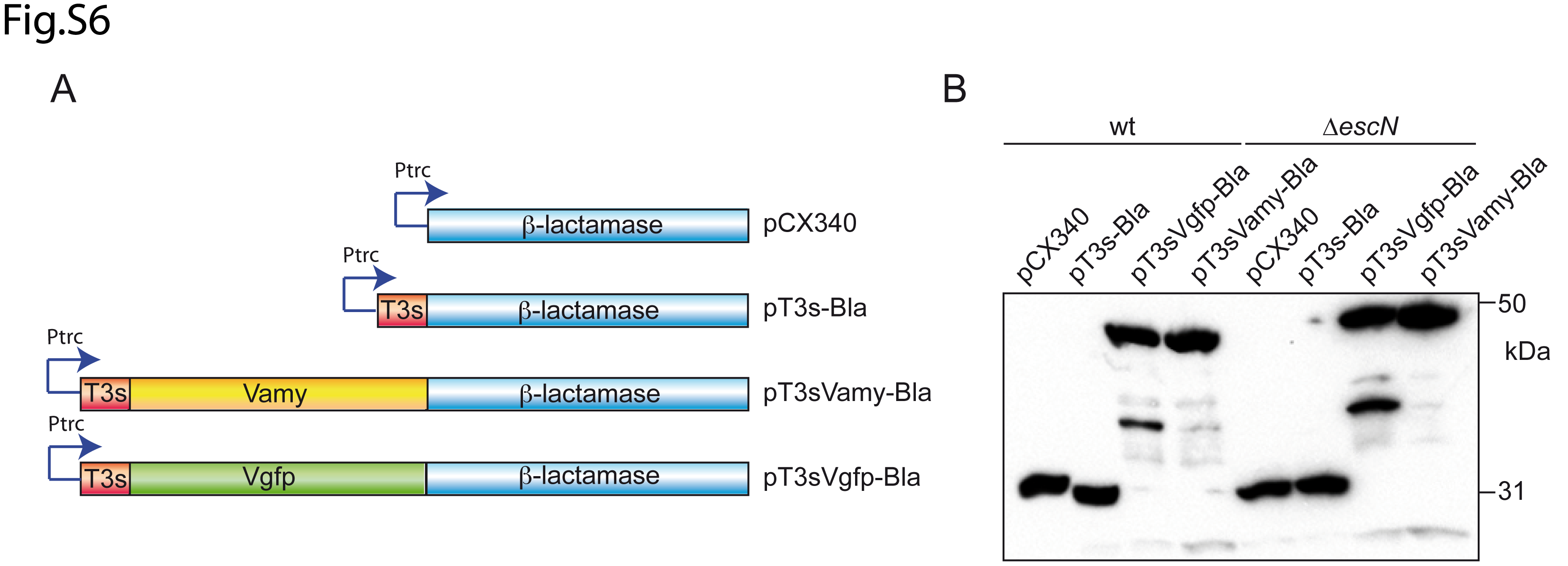

Supplement: Figure S6 — Expression of β-lactamase fusions in EPEC. A) Scheme of β-lactamase (Bla) gene fusions in plasmids pCX340, pT3s-Bla, pT3sVgfp-Bla and pT3sVamy-Bla. The position of Ptac promoter, T3 signal and VHH sequence, are indicated. B) Western blot developed with anti-β-lactamase mAb of whole cells protein extracts from induced EPEC wild type and ΔescN strains carrying the indicated Bla plasmid. Size in kDa of protein standards for SDS-PAGE is shown on the right. (TIF) [file pone.0015227.s006.tif]

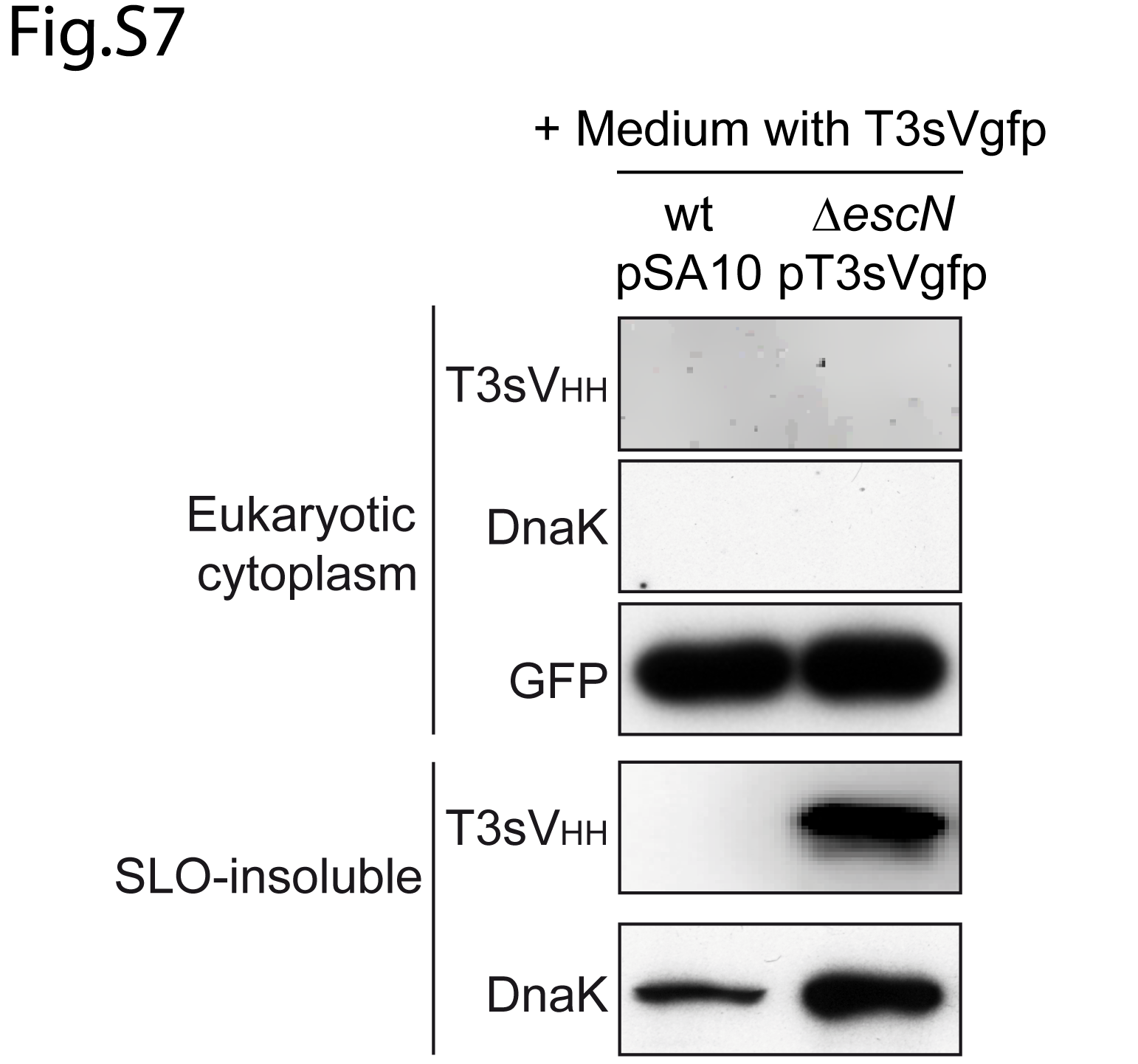

Supplement: Figure S7 — T3sVHHs do not enter into HeLa cells from extracellular media. Western blot of “eukaryotic cytoplasm” (top panels) and “SLO-insoluble” (bottom panels) protein extracts from infected HeLa cell cultures with EPEC wt/pSA10 and EPEC ΔescN/pT3sVgfp in which their extracellular media were replaced by medium containing T3sVgfp from induced EPEC wt/pT3sVgfp. Western blot of “eukaryotic cytoplasm” extracts are developed with anti-E-tag to detect T3sVHH, anti-DnaK to control the absence of bacterial contamination and anti-GFP mAb to test the efficacy of SLO pore formation. Western blot of “SLO insoluble” extracts are developed with anti-E-tag to show the expression of T3sVHH in bacteria and anti-DnaK mAb to control attachment of both strains to HeLa cells. (TIF) [file pone.0015227.s007.tif]

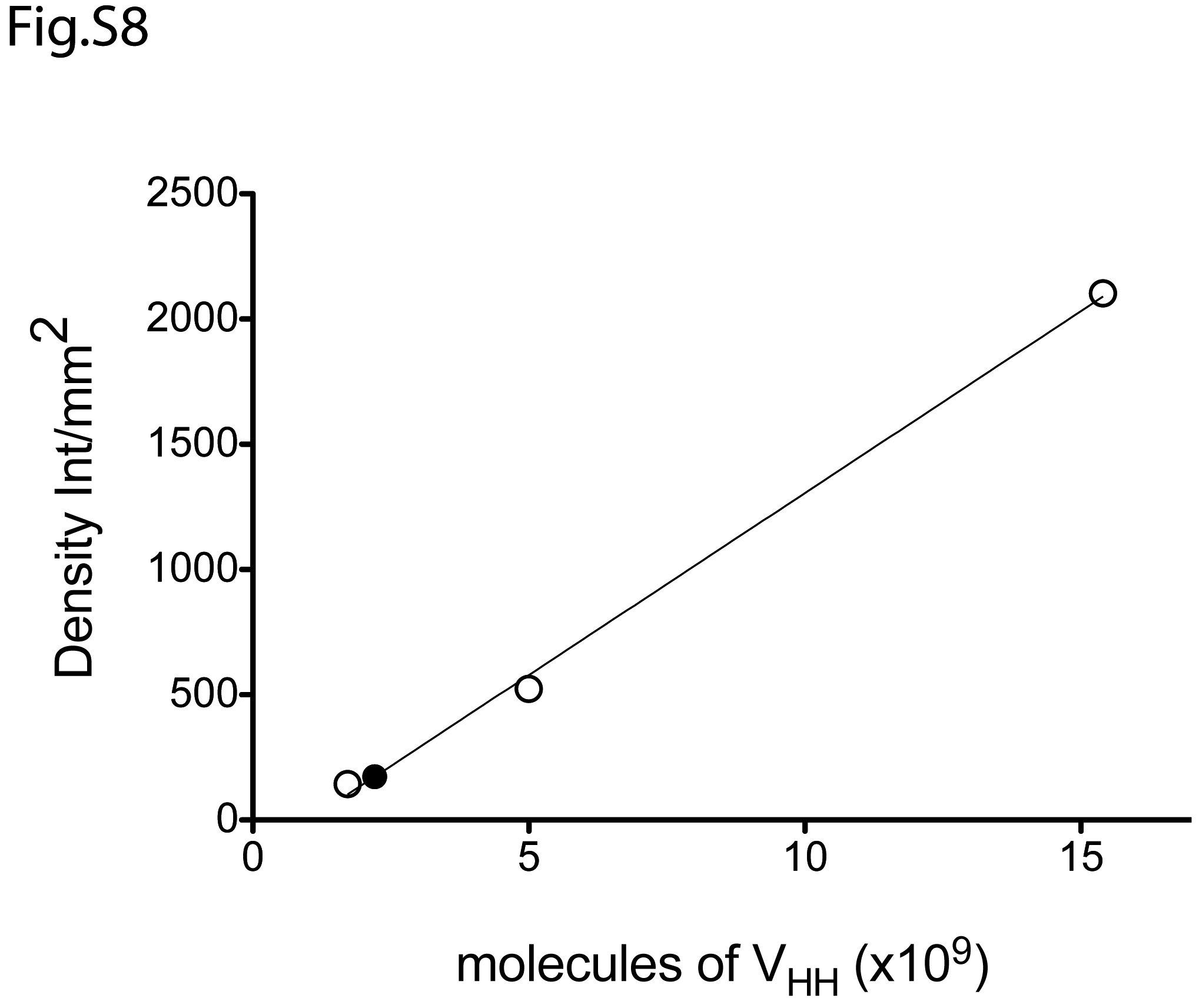

Supplement: Figure S8 — Quantification of T3sVHHs molecules injected. “Eukaryotic cytoplasm” protein extracts of pEGFPN1-transfected HeLa cells, infected with EPEC wt/pT3sVgfp, were analyzed by Western blot and the chemiluminiscence intensity of the protein band corresponding to T3sVHH determined by densitometry in a Chemi-Doc (Bio-Rad). The average intensity signal of T3sVHH corresponding to ca. 2300 HeLa cells from three independent infection experiments (closed circle) is interpolated to a stardard curve generated with the intesity of protein bands from samples of purified T3sVgfp of known concentration (open circles). (TIF) [file pone.0015227.s008.tif]
